# Telephone Games: How Issues With Common Citation Practices Established the Inverse Efficiency Score

**DOI:** 10.1027/1618-3169/a000666

**Published:** 2026-07-16

**Authors:** Heinrich R. Liesefeld, John Christie, Markus Janczyk

**Affiliations:** ^1^ Department of Psychology, University of Bremen, Germany; ^2^ Department of Psychology and Neuroscience, Dalhousie University, Halifax, NS, Canada

**Keywords:** slow science, metascience, questionable research practices, combined performance measures, speed–accuracy tradeoff

## Abstract

**Abstract:** In many experimental-psychological studies, either the speed or the accuracy of responses could be the primary dependent variable of interest. Often, there is no straightforward choice and this decision is particularly difficult if there are signs of variation in speed–accuracy tradeoff across conditions and/or participants. An apparently well-validated solution to this conundrum is the inverse efficiency score (IES). The general application of IES has never actually been endorsed in the cited sources and recent validation studies show that it does not serve well any of the major purposes to which it has been applied. Its popularity may even have hampered progress on the important methodological issue of how to best combine speed and accuracy measures. The present article traces the history of IES via a literature review and with the aid of contemporary witnesses. This exercise reveals a flaw in the academic culture that could be improved by increasing the value of either checking the original sources or instead being honest about the reliance on second-hand information. The story of IES also exemplifies how ignoring fundamental methodological problems makes practitioners resort to the most accessible solutions, often relying on a simplified appeal to authority and little else.

In many psychological experiments, participants are to make a choice between two alternatives on each trial. These simple experiments typically result in two performance measures: the speed and the accuracy of responses. It can easily be the case that each measure on its own does not seem to capture how independent variables impact performance. To illustrate, consider this outcome of a hypothetical experiment. After a psychological intervention, participants commit fewer mistakes in a cognitive test compared to a well-matched control group as measured in terms of proportion of correct responses (often shortened to proportion correct, PC) or proportion error (PE, which is 1 – PC). At first, this indicates that the intervention has successfully improved the underlying cognitive skill set and a paper titled “A new intervention improves cognitive performance” is submitted for publication. To the dismay of the authors, a reviewer requests to also see the pattern of mean response times (RTs) and they notice that the intervention group performs the task slower than the experimental group. This discrepancy – fewer errors but longer RTs in the intervention group – is a tell-tale sign of a speed–accuracy tradeoff effect.^1^ Thus, maybe the intervention group did not genuinely improve through the intervention, but the intervention merely caused them to perform the task more carefully, thereby avoiding mistakes at the cost of taking longer to complete the task. To determine which of the two groups performs better overall, a reviewer suggests to combine mean correct RTs and PCs by calculating the inverse efficiency score (IES) according to the formula:^2^

1 $${{IES}_{i,j}={\bar{cRT}}_{i,j}/PC}_{i,j}$$

The index *j* here stands for each condition and *i* stands for each participant in that condition, that is, IES is calculated separately and independently for each participant–condition combination. The authors are unfamiliar with this procedure and wonder whether they should follow the advice and calculate IES for their study.

Of course, IES does indeed combine measures of speed and accuracy. Furthermore, it does increase as mean RT increases and it decreases as PC increases so that higher values do indicate poorer performance and lower values indicate better. This fundamental property bestows a degree of validity to this combined performance measure. However, from that simple property, we cannot know whether it fulfills the more specific purposes it is used for. Consider that the equation is choosing how much RT and PC are being weighted, that is how strongly an increase in one or the other constituent measure affects IES and this weighting varies across performance and SAT levels (see Figure 3 in Liesefeld & Janczyk, 2019). Considering alternatives helps to understand how weak this justification is. There are an infinite number of conceivable combined performance measures that have the fundamental property of increasing when errors or RTs increase, such as subtracting PC from mean RT, taking mean RT to the power of PE, or taking the average of mean RT and PE. Any of these could be combined with transformations of the constituent measures, such as taking the base-10 logarithm of mean RT or an arctangent transform of PC, without losing the fundamental property. For some measures, linear transforms, such as whether the mean RT was in seconds or milliseconds, would also affect the results. These choices would not only affect the absolute value of the measure but also the relative difference between conditions, people, and blocks. To preface what comes, we will show that none of the cited fundamental sources for IES even claim through equations, simulation, or other validation that it fulfills its purported purposes; and that subsequent work shows that other combined performance measures are much better when used on simulated data for which the ground truth is known. Therefore, while IES is not completely invalid its limited face validity is only skin deep.

A literature search^3^ revealed two major reasons for using IES: (a) accounting for undesired variation in speed–accuracy tradeoff (SAT; e.g., variation in SAT across conditions, i.e., SAT effects, as in the above example), (b) obtaining a more comprehensive measure of task performance (e.g., because variation in SAT across participants might dilute an empirical effect), or both. Together, these motivate the application of IES in 52 of 63 papers that were published in 2023 and mention any reason for using IES. Regarding (a), extensive data simulations using the well-validated diffusion model (Ratcliff, 1978; Ratcliff et al., 2016; Schwarz, 2022) arrived at the conclusion that IES does not well-control for SAT effects and that there are much better alternatives (Liesefeld & Janczyk, 2019; see also Liesefeld & Janczyk, 2023, or Vandierendonck, 2017). In particular, Liesefeld and Janczyk (2019) observed significant IES differences and high effect sizes between conditions that were simulated to differ only in SAT. Regarding (b), the same simulations revealed that IES is mainly driven by mean RTs. In their Figure 3, Liesefeld and Janczyk (2019) show that, whenever responses are not extremely rushed, IES correlates highly with mean RT, but much less with PC. Both purposes (a) and (b) were best fulfilled by the Balanced Integration Score (BIS; Liesefeld et al., 2015):^4^

2 $${BIS}_{i,j}=z_{i,j}^{PC}-z_{i,j}^{\bar{cRT}}$$

where $z_{i,j}^{X}$ is the respective aggregated performance measure *z*-standardized across all participants and condition combinations.

Furthermore, an often-cited survey of empirical data asking whether IES “clarifies matters” pessimistically concludes that “It looks pretty much like every type of change is possible with the introduction of IES” (Bruyer & Brysbaert, 2011, p. 9; note that “change” here refers to the difference in pattern of significant effects between IES and the constituent measures). The best support we could find for IES was that it performs similar to other transformations in another area of psychological research, namely in individual differences work (Bakun Emesh et al., 2022). As detailed in the subsequent section, we find no articles awarding IES the value its general use suggests, but rather substantial evidence to the contrary.

To illustrate why this uncritical use of IES is problematic, consider the example in Figure 1. Panel (a) illustrates SAT curves at two performance levels based on a formula suggested by Wickelgren (1977; see also Liesefeld & Janczyk, 2023). Panels (b) and (c) show data simulated with a diffusion model (Ratcliff, 1978; see also Liesefeld & Janczyk, 2019, 2023). By varying the threshold parameter, we simulated a pure SAT effect resulting in longer mean RT and lower PE in Condition A compared to Condition B (Figure 1b). In these simulations, PE is below 10% as Bruyer and Brysbaert (2011) recommend for the use of IES. Still, IES does not cancel this effect but shows the same pattern as the mean RTs. BIS (Liesefeld et al., 2015; Liesefeld & Janczyk, 2019), by contrast, considerably attenuates the simulated SAT effect, resulting in average BIS values close to zero in both conditions. By varying the drift-rate parameter, we also simulated a “real” effect, that is, a difference in difficulty or ability between Conditions A and C (Figure 1c). This effect can be seen in all four performance measures.

**Figure 1:**
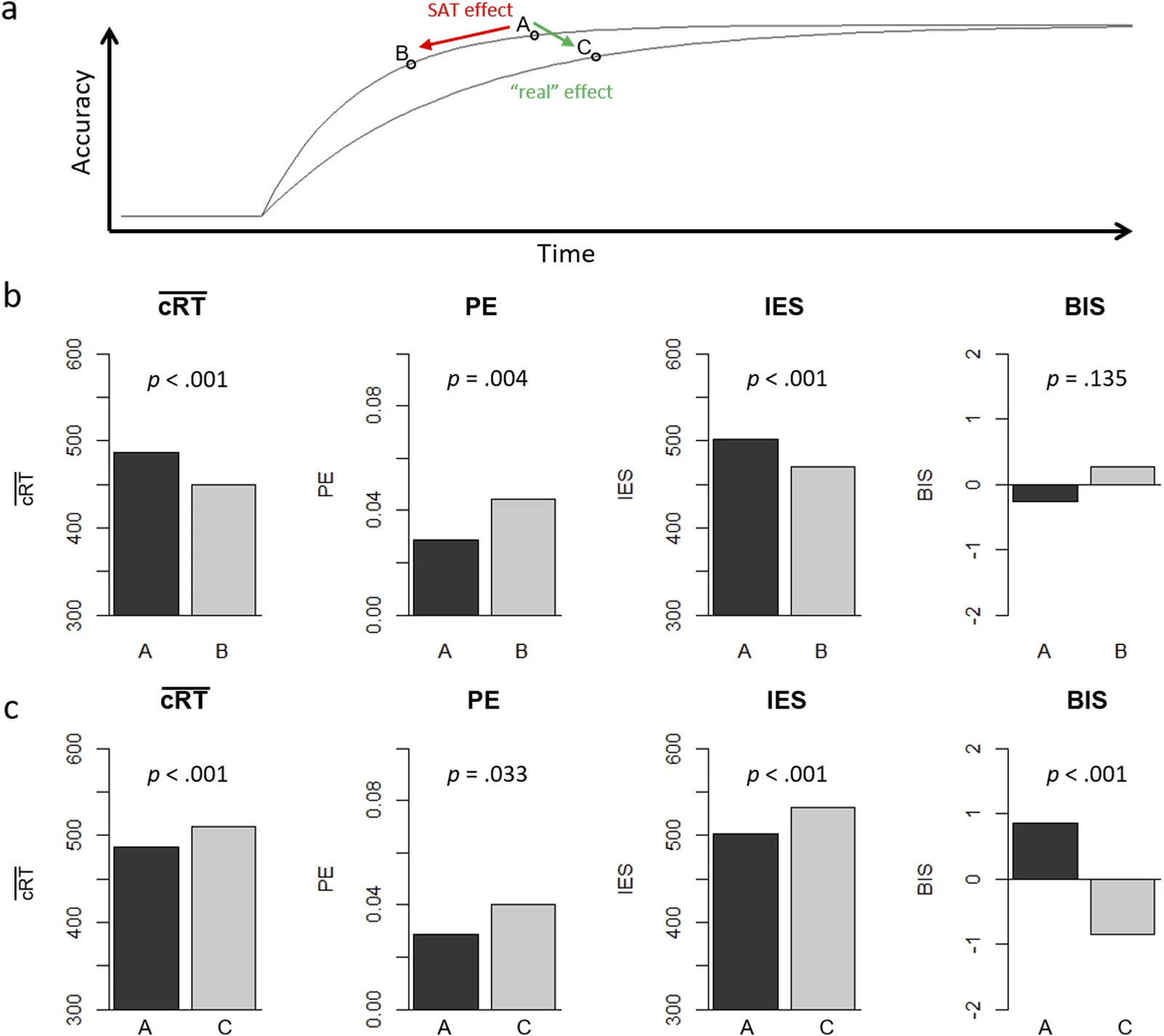
An illustration of SAT effects and “real” effects and how they affect various performance measures. (a) two different SAT curves corresponding to two different performance levels with relations between conditions A-C from Panels (b) and (c) exaggerated for illustration. (b) SAT effect between conditions A and B that lie on the same SAT curve, (c) “real” effect between conditions A and C that lie on different SAT curves. $\bar{cRT}$ = mean correct response time, PE = proportion error, IES = inverse efficiency score, BIS = balanced integration score. For BIS, higher values reflect better performance; for RT, PE, and IES, higher values reflect worse performance. ${{{IES}_{i,j}=\bar{cRT}}_{i,j}/PC}_{i,j};\;{BIS}_{i,j}=z_{i,j}^{PC}-z_{i,j}^{\bar{RT}}$, where $z_{i,j}^{X}$ is the respective performance measure for participant i in condition *j* z-standardized across participants and conditions.

We recognize that simulations showing that IES is not the best tool available to integrate speed and accuracy measures do not prove its use has harmed the field. Nevertheless, they do demonstrate that it is not a good solution and that there is very likely some amount of unnecessary harm that has been caused, and continues to be caused (discussed below in the section “What could happen?”). While recent work (summarized above) seems pretty damning for IES, we here sought to understand where the original support for the measure came from.

## The Origin of IES

In our exploration of the history of IES, we started in the usual places, looking at the references given as justification for it. Townsend and Ashby (1978) was given as a common source for IES and also was the earliest reference. Upon reading this dense book chapter on mathematical psychology, we realized that neither the term *inverse efficiency*, nor the equation for IES appear anywhere. There are various formulae of the form Q/T – “the total energy produced by a system, relative to the duration of time taken to produce it” (p. 212). These quantities are not directly observable and not suggestions of any applicable combined performance measure. Rather, the formulae are used to make a theoretical argument for understanding processing capacity as a combination of the amount of energy applied (Q) and the speed with which it is applied (T). Q/T might be the germ for IES since it is also a ratio involving time and might have convinced some researchers on a superficial read that Townsend and Ashby (1978) recommend IES. But it is clearly different from ${\;{\bar{cRT}}_{i,j}/PC}_{i,j}$ in many ways, not least that it is a theoretical quantity rather than an applicable performance measure. This was surprising to find, given that in 2023 alone, 19 scientific papers from various authors cite this chapter for precisely the application of a combined performance measure that is now typically referred to as IES. The same mistake, incorrect attribution of the IES formula to Townsend and Ashby (1978), can be found in an influential validation paper (Vandierendonck, 2017) and even in a paper written by the first author of the original chapter (Townsend, 2016; who cautions not to blindly trust in IES and highlights the need for validations, though). The most cited review on IES claims that: “Townsend and Ashby (1978) proposed the ‘inverse efficiency score’ (IES; see also Townsend & Ashby, 1983)” (Bruyer & Brysbaert, 2011, p. 6).

Given that Townsend and Ashby (1978) is not the source of the IES formula, we skimmed through the earliest publications that cited this work and found Akhtar and Enns (1989), who refer the reader to page 204 of Townsend and Ashby (1983). It makes sense that, if the germ of IES is in the 1978 paper, then Townsend and Ashby probably expanded and validated it in their book. Indeed, on that page the popular equation was found, and – as expected – it was related to the 1978 chapter and to the theoretical Q/T ratio. Yet, it was only a brief mention for a couple of pages and not the hoped-for elaboration and validation. On p. 204, we find:

What we need is some observable measure of the average energy consumed by the system over trials. […] We want this measure to increase with processing time and to decrease with improvements in accuracy, and we want to be able to compute it for each experimental condition. As a measure monotonically related to processing time, we can use mean RT. A desirable accuracy measure is often a statistic such as *d'*, the estimate of the detectability parameter from standard signal detection theory. Unfortunately, this parameter cannot be estimated in any single experimental condition. For example, in the target-absent conditions we can estimate the probability of a false alarm but not of a hit. An estimate of *d'* requires both of these probabilities. Thus we make the best of what we have. As a compromise, we might use the proportion of correct responses, P(corr), as our accuracy measure.

Next, we must decide what function of these two statistics we shall take as our estimate of the consumed energy. Townsend and Ashby (1978) suggest the ratio of the two, and for illustrative purposes, we adopt that notion here. Figure 7.12a shows a plot of $\bar{\text{RT}}/P\left(\text{corr}\right)$ versus display size that….

Thus, the ${{\bar{RT}}_{i,j}/PC}_{i,j}$ measure is only mentioned for illustrative purposes to make a theoretical point about parallel and serial search in this section of the book. It is not proposed as an ideal combination, but only as one among many possible quick ways to combine measures of speed and accuracy.

Although Townsend and Ashby (1983) can be cited for mentioning ${{\bar{RT}}_{i,j}/PC}_{i,j}$ as a combined performance measure and applying it in an illustrative figure, this was not the source of the IES term nor any validation or even endorsement. Perhaps IES was developed and validated in the first paper that used it and merely the inspiration came from Townsend and Ashby (1983). Therefore, we had a closer look at Akhtar and Enns (1989, pp. 321–322), which is the first empirical study using the measure:

What is needed is a combined measure of RT and errors, preferably one that measures the overall efficiency of performance without violating additive factors logic. Townsend and Ashby (1983) discuss the problem of analyzing RTs from errorful data and suggest using the measure: mean correct RT divided by the proportion of correct responses, RT/*p*(Correct). This measure has properties that are consistent with traditional intuitions of RT. For instance, increases in RT still result in increases in the new measure. Increases in errors also result in increases in the new measure. If no errors are made, that is *p*(Correct) = 1, then the mean correct RT stands unchanged. If accuracy is less than perfect, the new score increases in proportion to the number of errors. Townsend and Ashby (1983) refer to this measure as an “observable measure of the average energy consumed by the system” (p. 204).

It appears that Akhtar and Enns (1989) appealed to ${{\bar{cRT}}_{i,j}/PC}_{i,j}$ because they needed “a combined measure of RT and errors, preferably one that measures the overall efficiency of performance without violating additive factors logic” (p. 321). Subsequently, IES has become “a standard way to combine RT and accuracy measures of performance” and “a measure of processing efficiency that discounts possible criterion shifts or speed/accuracy tradeoffs” (Kennett et al., 2001, p. 466).

A comment from an anonymous reviewer on an earlier version of this manuscript provided a crucial hint to how one could misread Townsend and Ashby (1983): The reviewer argued that in the passage quoted above, Townsend and Ashby (1983) state that Townsend and Ashby (1978) had proposed the ratio of mean RT to PC. This would mean that Townsend and Ashby had misremembered their own work from 5 years earlier. This comment was at first perplexing to us, because they do not (incorrectly) attribute the specific formula to their 1978 work in this quote, but they (correctly) attribute the formula’s general form. After some contemplation, we realized that it is grammatically ambiguous what “the ratio of the two” refers to. From carefully reading Townsend and Ashby (1978), it appears that Townsend and Ashby (1983) refer to the ratio of two hypothetical constructs, namely processing time and accuracy, both of which still need to be operationalized. These are operationalized as mean RT and PC for illustrative purposes in the example by Townsend and Ashby (1983), but a thorough reading of Townsend and Ashby (1978) clearly suggests that these are poor placeholders. Even in the 1983 quote, they state that PC may not be the best operationalization of accuracy, but it is just a compromise available for their case. Crucially, “the two” could grammatically as well refer directly to mean RT and PC rather than to the underlying constructs (processing time and accuracy), hence the misreading.

The absence of an intent to suggest a practical application is also indicated by the “lack” of specification of IES by Townsend and Ashby (1983). This would also explain why so many versions of IES or derivatives exist in the literature that are justified by typical IES citations (viz. Bruyer & Brysbaert, 2011; Townsend & Ashby, 1978, 1983). In our 2023 sample (see Footnote 3), these versions include the inverse of IES (e.g., Kapadia et al., 2023; Zhao et al., 2023) and median RT as a speed measure (e.g., Kaur et al., 2023; Zhang et al., 2023); some studies also combine data across present versus absent conditions to calculate, for example, *d*’ as the accuracy measure (Duleme et al., 2023). Further below, we also discuss the question of whether RTs from incorrect trials are included into mean RT. The existing validation studies used PC and mean correct RTs as far as we can tell (Bruyer & Brysbaert, 2011; Liesefeld & Janczyk, 2019; Vandierendonck, 2017). Thus, overall, the most accepted way to calculate IES seems to be mean correct RTs divided by PC, separately for each participant *i* and each condition *j*.

In personal communication (June 6, 2025), Townsend reminded us that he and Ashby (then and now) strongly encouraged the development and utilization of quantitative tools more closely connected with each area of investigation. They in no way were promulgating this particular statistic in any sense that it had been vetted and demonstrated to be reliable and veracious with regard to any specific application. He stated that our attempt to clarify the origins of IES is appropriate and can hopefully lead to an even better science with or without this tool. He wishes this discussion could have begun much earlier. He further notes that he was unaware of IES’ gain in popularity for a while and was somewhat dejected that the deeper arguments, findings, and tools in Townsend and Ashby (1978) had been neglected. Having said this, after he became aware of IES, he remained open to the possibility that it might turn out to be useful for deciphering empirical phenomena and also did not feel responsible to either validate it or suppress its use. He draws a pertinent analogue in the decades of behavioral scientists’ using slopes in, first short-term memory search, then visual display search, to infer serial versus its polar opposite, parallel processing.

## Augmentation of ${{\bar{RT}}_{i,j}/PC}_{i,j}$

Likely, the formula for IES was used by Akhtar and Enns (1989) based on the misunderstanding that by illustrating ${{\bar{RT}}_{i,j}/PC}_{i,j}$ Townsend and Ashby (1983) were advocating its use. But there are many additional claims about IES that are typically ascribed to Townsend and Ashby (1978, 1983), but cannot be found in their work. These details need to be specified to apply ${{\bar{RT}}_{i,j}/PC}_{i,j}$ and to write about it. They include a name, the exact calculation rules, and preconditions for using ${{\bar{RT}}_{i,j}/PC}_{i,j}$. That Townsend and Ashby (1983) left these details unspecified is further indication that they did not intend to suggest the broad application of ${{\bar{RT}}_{i,j}/PC}_{i,j}$. If this is just an illustrative example, there is no need for any specifications. This created a problem for users of IES, who had to come up with the details themselves. They likely filled the blanks with their own ideas, community defaults, and probably further misreading of Townsend and Ashby (1978, 1983) as detailed in the following. This plethora of information on IES may have convinced other researchers that it was a well-vetted performance measure.

### Naming IES

For starters, where did IES get its name? No name for this measure is mentioned in Akhtar and Enns (1989), but Christie and Klein (1995) refer to it as an “efficiency score” and Murphy and Klein (1998) is the first published article to call it “inverse efficiency” or “inverse efficiency score,” interchangeably. Present author J. Christie, baptized it as “inverse efficiency” in his Master’s thesis (Christie, 1995), because it was inconvenient to refer to it as “$\bar{RT}/PC$.” He initially noticed that the equation is the same as the classic rate of information transmitted if information were held at a constant 1.0, recalling Hick (1952) and others. Early on, he called this “efficiency” (Christie & Klein, 1995) but that was short lived and he changed to “inverse efficiency,” since efficiency measures typically increase with increasing efficiency but ${{\bar{cRT}}_{i,j}/PC}_{i,j}$ decreases (see also Mullane et al., 2009, p. 325).

### Handling Variation in SATs

The most popular belief about IES was already mentioned above: it purportedly controls for SATs in some way. It is therefore often used when the effects of an independent variable on measures of speed and accuracy point in two different directions as exemplified in the introduction and Figure 1b (SAT effects). Akhtar and Enns (1989) do not mention any SAT. In using ${{\bar{cRT}}_{i,j}/PC}_{i,j}$ in his thesis, author J. Christie wrote: “In order to allow comparisons among conditions using a single measure that would incorporate any existing speed accuracy tradeoffs (of the sort just described) the data were converted to inverse efficiency (IE) scores (the mean reaction time divided by the proportion correct) following the recommendation of Townsend and Ashby (1983).” (Christie, 1995, p. 20).

Still, J. Christie’s thesis is not publicly available and therefore cannot have influenced other authors directly. The belief that IES would control for variation in SAT can be found more explicitly in Christie and Klein (1995, p. 549; note that “the RT difference” is a relative speed up):

Although the control and the experimental conditions at this SOA appear to differ only by degree (see Table 1), it must be noted that the RT difference in the control condition, in addition to being statistically unreliable, was accompanied by a significant drop in the accuracy […]. This demonstrates that the apparent similarity in trends in the control and experimental conditions may have been due to a speed-accuracy tradeoff in the control conditions.

Following the recommendation of Townsend and Ashby (1983), the data were converted to efficiency scores (RT divided by the proportion correct) in order to allow comparisons among conditions uncontaminated by any existing speed-accuracy tradeoffs.

Such contaminations of “real” effects with variation in SATs are not discussed in relation to ${{\bar{RT}}_{i,j}/PC}_{i,j}$ by Townsend and Ashby (1983). Rather speed–accuracy curves (see Figure 1a), that is, studying accuracy as a function of time within each condition and participant are a core theme in Townsend and Ashby (1978) and briefly mentioned in Townsend and Ashby (1983).

### Correct RTs

It is not self-evident what the “$\bar{RT}$” in the numerator refers to. RT research often takes only RTs from correct trials into account, because it is interpreted as the time needed to solve the task, whereas it is less clear what error RTs reflect. By contrast, SAT curves are derived from correct and incorrect RTs, because they show the accuracy level (PC) as a function of the amount of time invested. In the above quote, Akhtar and Enns (1989) use only RTs from correct trials (according to their text; this is not reflected in their printed formula). There is no hint to using only correct RTs in the purported source (Townsend & Ashby, 1983). J. Christie had used correct RTs only, but this is not reported in Christie and Klein (1995) nor in Christie (1995). This analysis choice was not mentioned because it is so common to use only correct RTs that for many, including Christie and Klein, this is tacitly assumed if not specified otherwise. Not every researcher is aware of this practice though. Maybe because of this ambiguity, there is some variance in the literature, with regard to the exclusion of error trials. Of 66 papers that use IES in 2023 *and* report a formula, 16 report using only correct RTs, whereas 50 papers specify neither inclusion nor exclusion of incorrect RTs. Given the abovementioned community standard, we still assume that most IES reports include only correct RTs.

### Positive and Linear Relationship Between RTs and Error Rates

Bruyer and Brysbaert (2011, p. 11) write that “Townsend and Ashby (1978, 1983) warned that the IES only works when there is a positive correlation between RT and PE: the IES transformation should be used only if high and linear correlations are evidenced.” The same claim can be found in other sources (e.g., Hughes et al., 2014). Although it remains slightly unclear what exactly is meant (a correlation across participants, conditions, trials, or any combination thereof), most interpretations of this requirement are not reasonable, and the purported sources do not contain this advice. Nowhere do Townsend and Ashby (1983) state that ${{\bar{RT}}_{i,j}/PC}_{i,j}$ requires “a positive correlation between RT and PE.” Because Townsend and Ashby (1978) do not even mention ${{\bar{RT}}_{i,j}/PC}_{i,j}$, they cannot be the source for this requirement. The source for this criterion can be found in Akhtar and Enns (1989, p. 323):

An important assumption underlying the use of this measure is that it is a linear transformation of RT. This assumption is especially important for the present experiment because the additive factors method assumes at least an interval scale of measurement. Townsend and Ashby (1983) point out that this assumption can be tested by examining the correlation between RT and errors. If the correlation is high and the relation is linear, then one measure can be treated as a linear transformation of the other measure.

“RT and errors” refer to mean (correct) RTs and PE per condition, as can be seen from how Akhtar and Enns (1989) examine this purported assumption: “Mean RTs were positively associated with mean percentage errors for the 18 Orienting × Filtering × Session conditions for each age group in the present experiment, *r*(16) = .69, .76, .88, and .82, for 5, 7, 9, and 24 year olds (all *p* values < .01).” (p. 323). The 16 degrees of freedom indicate that 18 data points went into each correlation, which apparently come from the 18 within-participant conditions per age group.

The quotes also show that Akhtar and Enns (1989) did not require a positive correlation, but observed one. While “high” can be interpreted as positive, it could also have been intended as “extreme,” whether positive or negative. Requiring a positive correlation would be at odds with the claim that IES can control for SATs (see the respective section above), which would typically result in negative correlations between mean RT and PE across conditions. In fact, observing a positive relationship between mean RT and PE is the very situation in which, according to our experience, most researchers (and reviewers) see no need for combining the two measures; if both measures point into the same direction, interpretations of the two performance measures support each other rather than being in conflict.

Furthermore, the requirements that ${{\bar{RT}}_{i,j}/PC}_{i,j}$ be a linear transformation of RT and that this could be tested via a correlation between ${\bar{RT}}_{j}$ and *PC*_*j*_ are problematic for a number of reasons. First, if there is any relationship between RT and PC (or PE), it must be nonlinear already because PC has a ceiling at 1, whereas RT has no in-principle upper bound. In a given experiment, mean RT and PC could only be linearly related due to sampling error or if a small subset of the function is sampled, because any curve is locally linear and any two points can be connected by a straight line. This does not reflect the relation between mean RT and PC in any representative sample. Therefore, this assumption cannot be expected to be reasonably well met even if it is only a sample property. Second, whenever PC differs between participants and conditions (as it usually does), IES is quite obviously not a linear transformation of mean RT, even if its constituent measures PC and mean RT are perfectly correlated (this was also noted by Bruyer & Brysbaert, 2011). This is because a linear transformation is achieved by adding a constant or multiplying by a constant factor, or both. The 1/*PC*_*i,j*_ in the IES formula would only be a constant factor if PC was constant across conditions and participants (i.e., *PC*_*i,j*_ = *PC*; in which case, the correlation between mean RT and PE as calculated by Akhtar & Enns, 1989, would be zero).

We further checked Townsend and Ashby (1983) to see if we could derive an interpretation that supports the linearity requirement. Townsend and Ashby (p. 203) do argue that the issue of looking at accuracy becomes important “when accuracy losses are consequential, and especially when these losses are correlated in some way with RT.” They do not note which type of relationship or direction it should be, just some relationship could make it more important to look at integrated RT and accuracy. It is possible that Akhtar and Enns interpreted “correlated in some way” as a Pearson correlation and the linearity implied. The only place linearity is mentioned by Townsend and Ashby (1983) is, “The linearity of the curves [relating ${{\bar{RT}}_{i,j}/PC}_{i,j}$ to display size] will not be affected so long as the accuracy decrements are linearly related to display size.” (p. 204). This is not the linearity that Akhtar and Enns argued is a requirement for calculating ${{\bar{cRT}}_{i,j}/PC}_{i,j}$. This is all in keeping with Townsend and Ashby only using ${{\bar{RT}}_{i,j}/PC}_{i,j}$ in one case for illustrative purposes.

The belief that IES requires a positive linear relationship between RT and PC might be reinforced by another misreading of Townsend and Ashby (1978). On p. 220, they suggest using the slope of the linear function relating (*d*’)^2^ to RT as a measure of processing power or capacity. This potential combined performance measure is completely different from IES and has, to our knowledge, never been applied so far (but see Dennis & Evans, 1996, for similar ideas). It is also not just a transformation of PC, because this approach requires the researcher to experimentally manipulate the SAT or make use of the spontaneously occurring covariation in RTs and accuracy (conditional-accuracy or SAT functions; Townsend & Ashby, 1983, 1978, refer to these as “speed–accuracy curves”). It can therefore not be the foundation for IES, but might indeed be a reasonable approach to control for SATs and therefore a competitor of IES. This approach, as they point out, presupposes that the relationship is indeed positive and linear. This slope must be positive, because if variance in these measures is purely due to variation in SAT, taking more time (longer RTs) must go along with better performance (higher (*d*’)^2^). It must be a linear relationship, because otherwise it would not be valid to fit a linear function. It is possible that someone trying to just scan Townsend and Ashby (1978) for support for IES could have latched onto this. But it does not have anything to do with IES and certainly not the requirement that mean RT and PE be positively correlated since that is the opposite of an SAT effect. Furthermore, in contrast to what Akhtar and Enns (1989) had tested, the positive and linear relationship must apply within a typical experimental condition rather than across conditions.

### At What Level of Accuracy Has IES Been Recommended?

One elaboration for which the source is clear is that IES should be used only for high accuracy levels. Based on their reanalyses of published empirical data, Bruyer and Brysbaert (2011) recommend that “*IES* is better not used when *PC* < .90” (p. 10; see also Vandierendonck, 2018). This recommendation is directly opposed to Townsend and Ashby’s (1978) assessment that PC can be ignored and analyses can focus on just RTs when PC > .90 (but see Wickelgren, 1977, and Figure 1 above). Townsend and Ashby (1978) state that “it may be that error rates up to about 10% (with error rates greater than 10%, Pachella, 1974, began finding slope changes) do not greatly influence the comparison process and therefore may be safely ignored when modeling this stage” (p. 224) and the same thought (without putting a number on it) can be found in Townsend and Ashby’s (1983) preface to their “suggestion” of ${{\bar{RT}}_{i,j}/PC}_{i,j}$. Accordingly, Akhtar and Enns (1989) included a high error rate as one of their reasons for using ${{\bar{cRT}}_{i,j}/PC}_{i,j}$ and so did many later applicants of IES. Thus, Bruyer and Brysbaert (2011) advise against using IES for exactly the situation where others think that a combined performance measure could be useful.

Townsend and Ashby’s (1983) argument that it is only necessary to take PE (or PC) into account when PE is high was grounded in the logic that, if PE is low, RTs contain all the necessary performance information. The IES transform would have its largest effects when PE is high and hence PC is low, because PC asymptotes at 1 and it goes into the denominator. Consider as an example, dividing by 0.95 versus 0.65, which results in a 5% versus 54% increase. Bruyer and Brysbaert’s (2011) finding that IES is best when PC is high may be considered as another piece of evidence that it is potentially not a good transform since very low PE (i.e., high PC) will tend to cause it to have very little effect.

### A Telephone Game

All these observations reminded us of a telephone game. As can be seen in our examination of the linear-positive relationship claim, Townsend and Ashby (1983) argued that a correlation between RT and PC renders it more important to consider both measures. Akhtar and Enns (1989) make a high Pearson correlation (i.e., a linear relationship) a requirement and further report that the correlations in their study were positive. Bruyer and Brysbaert (2011), or others before them, conclude from these, or maybe from a misreading of Townsend and Ashby (1978), that it is a requirement that mean RT and PE must be positively correlated. Bruyer and Brysbaert is an understandable interpretation of Akhtar and Enns (1989) which is further an understandable interpretation of Townsend and Ashby (1983). But the first and the final element in the chain are almost unrelated, because some information is lost and other information is added along the way. Similarly, the practice of using only correct RTs was added by Akhtar and Enns (1989), because analyzing only correct RTs is common in RT research. A name was added by Christie (1995) and first published by Murphy and Klein (1998), because this makes it easier to write about it, but they are not credited with it. Christie and Klein (1995) originated the claim that IES controls for SAT based on present author J. Christie’s reading of Townsend and Ashby (1978), who are indeed deeply concerned with SATs. Townsend and Ashby’s (1983) notion that combining measures of speed and accuracy was relevant when accuracy is low got lost along the way (after Akhtar & Enns, 1989), so that Bruyer and Brysbaert (2011) could come up with the exact opposite recommendation to use IES only when accuracy is high.

## The Growth and Impact of IES

### Rise in Popularity

In between Akhtar and Enns (1989) and Christie and Klein (1995), we found only one paper (D’Aloisio & Klein, 1990) and one thesis (Randolph, 1994) employing IES, although this is admittedly difficult to search without the IES name and we might have missed some. In general, use of the measure seems to have been mostly confined to people working with Raymond Klein during the 90s (Christie & Klein, 1995; D’Aloisio & Klein, 1990; Murphy & Klein, 1998).

In the early 2000s, researchers working with Charles Spence (who had been a visiting scientist in Klein’s laboratory in the late 90s) started to use IES extensively (Kennett et al., 2001; Pavani et al., 2002; Spence, Kettenmann et al., 2001; Spence, Kingstone et al., 2001; Spence, Pavani & Driver, 2004; Spence, Pavani, Maravita et al., 2004; Spence, Shore et al., 2001). Spence continued to use IES frequently in the late 2000s (Chan et al., 2005; Igarashi et al., 2007, 2008; Kitagawa et al., 2005; Kitagawa & Spence, 2005; Röder et al., 2007), but now other researchers also started employing the measure (e.g., Collignon et al., 2008, 2009; Graham et al., 2006; Horowitz & Wolfe, 2003; Kimchi & Peterson, 2008; Kunar et al., 2007; Setti et al., 2009).

It is somewhat difficult to track the evolution of the use of IES, because it is common practice not to cite any source for established measures and statistical procedures (such as *t* tests, ANOVAs, or outlier criteria), so that a large share of IES uses will go unnoticed when just looking at citation counts. Furthermore, most papers citing any source for IES do not cite the original Akhtar and Enns (1989) source nor any of the 1990s papers from Raymond Klein’s group. Townsend and Ashby (1983) is often cited for IES, but it is a large book that has been cited for many purposes (75% of citations of the 1983 book in 2023 were for IES, though). A compromise solution is to look at the citations of Townsend and Ashby (1978), which according to our detailed review of publications of the year 2023 was cited for IES in 28 of 31 cases (90%) and might therefore serve as a proxy to quantify the popularity of IES. Citations of this chapter over time compared to an index of the total number of papers in experimental psychology are displayed in Figure 2. Eyeballing the figure indicates that part of the development of citation rates (the levelling off after 2015 in particular) can be explained by the general popularity of experimental psychology. However, the sudden rise in citations around the year 2013 (a 275% increase from the average across the three years before to the three years since 2013) surpasses that of experimental psychology (a 31% increase between the same time ranges) and indicates to us that a paper published after 2010 or so is key to the rise in popularity of IES, or at least for the practice of citing Townsend and Ashby (1978) for this purpose. We can only speculate on the cause for this rise, but Bruyer and Brysbaert’s influential IES review was published on February 1, 2011. Assuming a year for this article to get widely noticed and another year between data analysis and final publication of a research paper, the review could be the cause for the rise in citations of Townsend and Ashby (1978) and might have popularized IES as a performance measure. Ironically, Bruyer and Brysbaert did not even recommend the use of IES, but draw a much more nuanced conclusion that, if taken seriously, would discourage most researchers from using IES: “Although we set out with high hopes that the *IES* measure could be a better and a more concise variable than *RT* and *PE* to convey the findings of a cognitive psychology experiment, our analyses have shown that the ‘blind’ use of *IES* as *DV* is likely to lead to interpretation problems.” (p. 11). But at least this review is something one can cite.

**Figure 2:**
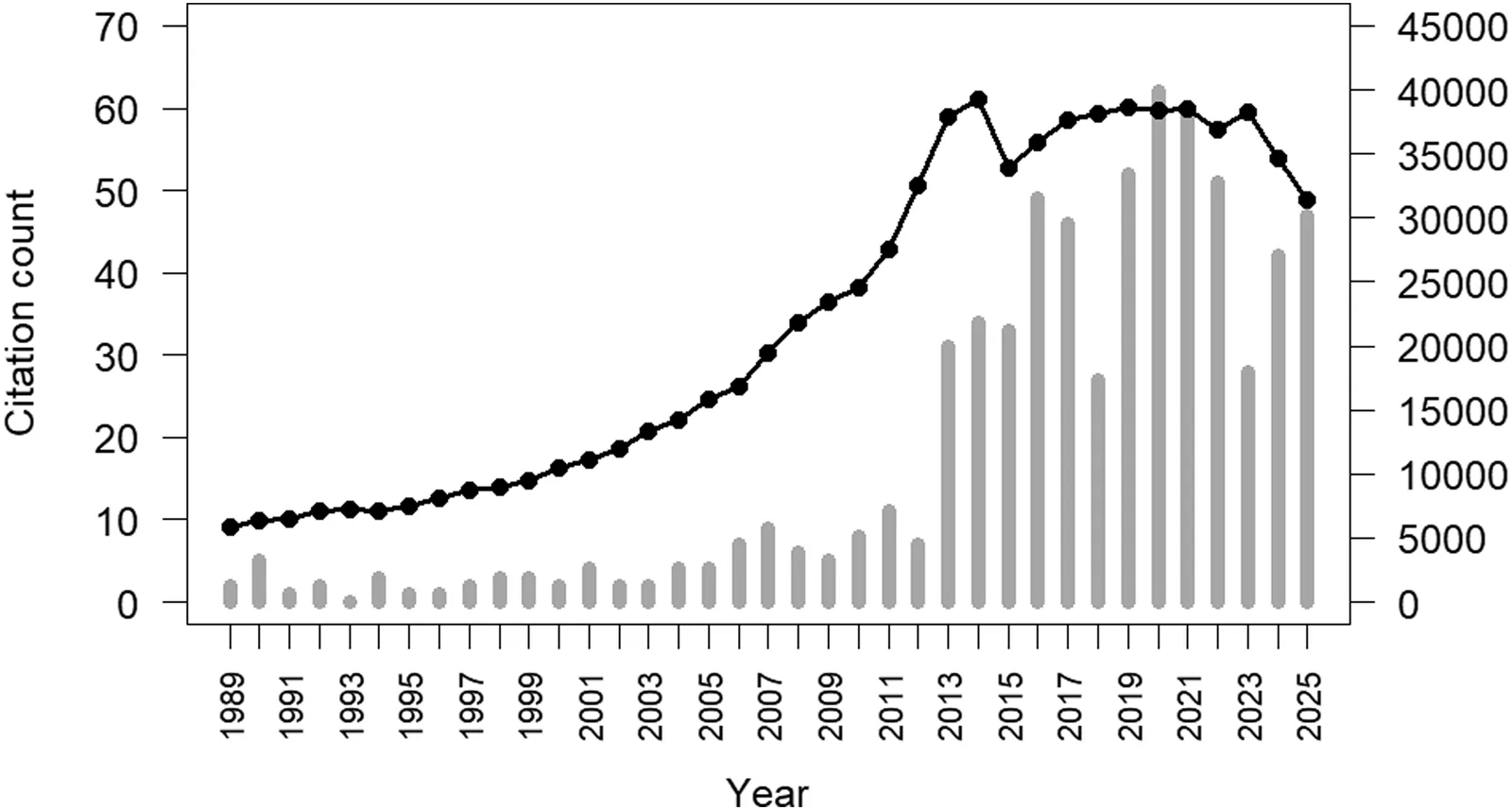
Rise of citations of Townsend and Ashby (1978). The bars and left axis show the citations of Townsend and Ashby (1978) and the line and right axis show the approximate number of search results for “experimental psychology” for comparison. Google Scholar on February 27, 2026. Note that this count includes duplications and papers that we were not able to access (nonelectronical books and theses).

Bruyer and Brysbaert (2011) might have contributed to misciting Townsend and Ashby’s works, as they claim “Townsend and Ashby (1978, 1983) proposed the inverse efficiency score (*IES*) as a way to combine both measures and, hence, to provide a better summary of the findings.” (p. 5). However, the Bruyer-and-Brysbaert review is not the source for misciting Townsend and Ashby (1978), because the practice of citing this chapter had already been firmly established in 2010 (i.e., before publication of Bruyer & Brysbaert, 2011). In 2010, 4 of 8 papers cite Townsend and Ashby (1978) for IES (50%) and 15 of 23 papers do so in 2013 (65%). Even though it is evident that they cannot have taken IES from Townsend and Ashby (1978), from the papers published in 2013 using IES none cites Akhtar and Enns (1989) and only three cite Townsend and Ashby (1983) in addition. Only one of these papers cites Bruyer and Brysbaert (2011) along with the two Townsend-and-Ashby papers (Lao et al., 2013). This indicates that it is common to reproduce information found in a secondary source and cite the primary source without checking the primary source and without citing the secondary source. Such practice would explain the telephone-game outcomes reviewed above.

### What Happened? Some Speculations

In sum, “the oldest and most frequently used measure that integrates RT and PE” (Vandierendonck, 2017, p. 654) was: (1) never proposed at all in one of the primary cited sources (Townsend & Ashby, 1978); (2) the equation occurs in the other primary cited source, Townsend and Ashby (1983), but it was not endorsed for its typical use, or validated; (3) criteria for its use have arisen from misinterpretations or confusions with other deliberations by Townsend and Ashby; and (4) recent efforts to validate this measure have been disappointing (Bruyer & Brysbaert, 2011; Liesefeld & Janczyk, 2019; Vandierendonck, 2017). This measure even received a name and there are established (but sometimes contradicting) rules of how and when to apply it. This richness of information on IES is suited to convince researchers that it is a well-validated method.

We can only speculate on how this could happen, but believe that the main reason was a combination of time pressure and an unfulfilled need for a performance measure that reasonably combines speed and accuracy and/or handles undesired variation in SAT. One might call the resolution of this predicament a *researcher speed–accuracy tradeoff*. Bruyer and Brysbaert (2011, p. 5) provide an illustrative example for why such a pragmatic tradeoff might be desired from the researcher’s perspective who tries to make the best of the already collected data:

First, authors check whether the conclusions based on *PE* and *RT* go in the same direction, or whether there is evidence for a speed-accuracy trade-off. In the latter case, the conditions with faster responses have higher error rates. In such a situation it usually is impossible to reach a convincing conclusion. […] The situation would be simplified if *PE* and *RT* could be integrated into a single *DV*, which appropriately weighs the impact of speed and accuracy.

Another pragmatic reason to use combined performance measures is aptly expressed by Murphy and Klein (1998, p. 1107):

Because some subjects might show the effects of interest in speed of responding while others might show them in accuracy, we tested the main predictions using a composite measure. Reaction time divided by proportion correct […] is just such a composite measure.

The first reasoning aims at accounting for systematic across-conditions variation in SAT, whereas the second reasoning aims at accounting for a participant-by-condition interaction in SAT (some participants commit more errors in the condition for which they would otherwise take longer).

After a group of mutually interconnected researchers started using IES for these pragmatic reasons (Akhtar & Enns, 1989; Christie & Klein, 1995; Kennett et al., 2001; Murphy & Klein, 1998; Pavani et al., 2002; Spence, Kingstone et al., 2001; Spence, Kettenmann et al., 2001; Spence, Shore et al., 2001; Spence, Pavani & Driver, 2004; Spence, Pavani, Maravita et al., 2004), other researchers read their papers or implemented their advice to use IES. Unfortunately, many of them shied away from reading (and/or trying to understand) the mathematically advanced Townsend and Ashby (1978, 1983) works. The difficulty of these works for the typical nonmathematical psychologist probably had two effects on the reader just looking for an easy solution for their speed–accuracy problem: (a) it seems to provide credibility for IES and (b) it discourages users of IES to actually read the texts. This reading seems dispensable because of the perpetuated impression that IES is a validated measure. As it is a merely methodological point to most researchers, they simply trust in the assessment of the sources they got it from. That Townsend and Ashby are published in book format increases the effort to get the texts via the usual sources. The books had not been digitized for quite some time and were therefore not easily obtained and searched for the relevant information. Probably most important is the confident tone with which IES is often described as a validated measure and the sheer number of studies using it that can be taken as its apparent support.

Interestingly, this confidence expressed by the academic community might even have convinced one of the original authors. This could happen because this telephone game has stretched over multiple decades and human memory is fallible. In a commentary on a study that uses IES, Townsend (2016) writes that Townsend and Ashby (1978) “propose as a rough and approximate method of cancelling out speed–accuracy tradeoffs, the statistic (employing Kristjansson’s terminology) inverse efficiencies (IES) = M RT/(1–M Error Rate)” (p. 3). It is fair to acknowledge that authors who cite Townsend and Ashby (1978, 1983) after having read this commentary are operating with some endorsement from Townsend. We are open about the question whether such (rather indirect) endorsement by one of the original authors frees researchers from the obligation to carefully check the sources for themselves, in particular as Townsend (2016) does not provide any empirical or theoretical support for the validity of IES. But if they consider this as the crucial piece of evidence that IES is valid, these authors must cite the commentary to support their use of IES. None of the seven papers that cite this commentary (according to Google Scholar on March 3, 2026) does so for IES. Furthermore, even without this interesting turn of events, the story of IES until 2016 is illustrative of unfortunate reading and citation practices in psychological science. If Townsend himself was indeed led astray by the convincing tone with which IES is ascribed to his work (by Kristjánsson, 2015, and many others before him), this anecdote underscores the impact of the telephone-game phenomenon investigated in the present paper.

### What Could Happen?

Why would it matter if IES (the whole complex, including formula, name, and preconditions) was not derived from the theoretical work of Townsend and Ashby (1978, 1983), but instead emerged from misinterpretations of their writings by others? An IES proponent might argue that it has been proven useful in hundreds of studies. Our answer hinges on what “useful” means here. The two reasons to use IES revealed by our literature research are (a) that IES accounts for presumed variation in SAT and (b) that it is a more comprehensive measure of task performance. Both assumptions are mistaken (Liesefeld & Janczyk, 2019) and our analysis of the origins of IES shows that – other than personal intuitions and the intuitions of other researchers before – there was never any reason to believe that IES would fulfill these purposes. Despite exclusively negative evaluations (Bruyer & Brysbaert, 2011; Liesefeld & Janczyk, 2019; Vandierendonck, 2017), IES is frequently used to the present day.

This persistence indicates that IES must be “useful” in another sense. Many published reports of IES point out that mean RT and PC patterns were contradictory and IES is used to decide which pattern is true and this scenario also takes center stage in the influential IES review by Bruyer and Brysbaert (2011; see the above quote for example). Using IES then is a backup strategy to make something out of otherwise difficult to interpret (and publish) data. Based on our own experiences in various roles, we believe that this strategy is often used to defend a study against a reviewer pointing out the contradictory pattern as a sign of an SAT effect or explicitly encouraged by reviewers noticing such a pattern. It might often be more a ritual rather than a serious attempt to arrive at the truth, just like null-hypothesis significance testing often is (Gigerenzer, 2004).

From this perspective, IES is a pragmatic approach to convince reviewers and editors that data are interpretable despite contradictory mean-RT and PC patterns. Given that open-peer review is not (yet) well-established in the psychological research community, this convincing usually happens behind the scenes. But sometimes, it happens in the open. For example, Kristjánsson (2015) observed that mean-RT-based visual-search slopes were affected by the required (search unrelated) response and therefore concluded that these slopes cannot be used to estimate search efficiency or distinguish search modes, in contrast to the common practice in research on visual search. In a comment, Wolfe (2016) calls this conclusion into question and highlights that in Kristjánsson’s data “Higher errors tend to be associated with lower mean RTs on the correct trials (a classic speed–accuracy tradeoff)” (p. 4). Kristjánsson (2016) counters by demonstrating that “the IES transform does not affect the patterns in the results in any fundamental way” (p. 2). We hasten to add that Kristjánsson then takes pains to warn the reader that IES might not be perfect, including the possibility that other combinations of mean RT and PC might be better and that the validity of a combined measure might depend on the weighting of its constituents.^5^ However, often IES is used more uncritically when a solution to handle or protect against reviewer critique in terms of discrepancies between mean RT and PC patterns is sought. Furthermore, the more careful and nuanced interpretation is often lost when space (word count) or time (to read the full paper) is limited, as for example in Kristjánsson’s (2016) abstract: “But when reaction times and errors are combined in one score (inverse efficiency) to sidestep speed accuracy tradeoffs, slope differences still remain.”

This is not to say that Kristjánsson’s (2015, 2016) conclusions are incorrect. It has been argued that set-size slopes should not be used to estimate search efficiency or distinguish between search modes (Liesefeld & Müller, 2020; Michaelsen et al., 2024; Townsend, 2016). Generally, whether IES is a good performance measure and whether conclusions drawn from the IES pattern happen to be true is of no relevance here (see instead Bruyer & Brysbaert, 2011; Liesefeld & Janczyk, 2019). Rather, the problem is that IES has been employed in hundreds of studies without any theoretical or empirical validation. Rejecting criticism like that by Wolfe (2016) based on a (re-)analysis with IES is invalid independent of the truth of the conclusions, because IES has never been (successfully) validated for resolving discrepancies between mean RT and error patterns. No combined performance measure perfectly cancels the SAT confounds that cause these discrepancies, but the currently most promising solution is BIS (Liesefeld et al., 2015; Liesefeld & Janczyk, 2019, 2023; see Figure 1).

There is another issue with how IES and other combined performance measures have been used. Because these measures are often utilized after other analyses have failed to confirm a hypothesis (Janczyk et al., 2023, is a recent example from the present team of authors where the effects on mean RT and PC were post hoc considered jointly by using IES as their combine), it can be considered an additional researcher degree of freedom (Simmons et. al., 2011). Trying new analyses after the original analyses have failed has been shown by Simmons et. al. (2011) to inflate the probability of false positives in declaring findings statistically significant. Thus, a typical way that IES and other combined performance measures are used is itself often problematic.

That IES was believed to have come from the well-grounded theoretical framework laid out by Townsend and Ashby (1978, 1983) likely was a major reason why it has been uncritically treated as if it had been validated. It does not seem overly implausible that validation efforts (Bruyer & Brysbaert, 2011; Liesefeld & Janczyk, 2019; Vandierendonck, 2017) would have been undertaken earlier and/or IES would have been interpreted more carefully if its provisional nature had been highlighted in Akhtar and Enns (1989) and subsequent studies. Furthermore, that IES was widely considered a validated measure has likely hampered the development and/or popularization of other combined measures that better handle SAT confounds and provide a more balanced integration of speed and accuracy (Liesefeld & Janczyk, 2019). “Establishing” IES as a way to handle SATs has yielded an ostensible solution and thereby aborted important discussions prematurely. Importantly, the use of inappropriate dependent measures is an issue that is not solved by otherwise effective methods to improve psychological science, such as replications (Rotello et al., 2015).

## Broader Implications for Academia

Instead of carefully checking and understanding the primary sources, most users of IES have blindly relied on the assessment of previous researchers, participating in what is essentially a telephone game. What is more, many of them did not disclose where they got the idea of and instructions for using IES from or did not make this sufficiently clear, thus obfuscating the transmission history. Miscitations in psychological science have been acknowledged and studied recently as an underexamined questionable research practice. In their sample of 3,347 citing claims from 89 articles, Cobb et al. (2024) found that 9.5% completely mischaracterized findings from prior empirical research. The story of IES is one in-depth case example that complements this work. Another popular example for bad citation practices has been provided by Gould (1988, as cited in Wynn, 2010), who traces back the textbook myth that the early horse resembled a fox terrier to a mistaken statement by a director of the American Museum of Natural History made in 1904. The history of IES is of particular relevance because of the involvement of so many people who incorrectly attribute IES to Townsend and Ashby (1978, 1983) and others who miscite Bruyer and Brysbaert (2011), Liesefeld and Janczyk (2019), among others, for support of its use. Citing these articles as the justification to use IES, would fall into the worst “inaccurate” category by Cobb et al. (2024) and the history of IES illustrates how “miscitation has the potential to proliferate into erroneous consensus around a topic, harm theory-building efforts, and even influence evidence-based practice” (Cobb et al., 2024, p. 300; see also Gigerenzer, 2004).

Even though we present a single case study, the sheer number of researchers who miscited the sources shows how serious the problem is. As so many colleagues apparently committed the mistake of ascribing IES to Townsend and Ashby (1978) and/or to take the illustrative example in Townsend and Ashby (1983) as motivation to apply the formula to their data, this is a great example to illustrate an unhealthy aspect of the current academic culture and the potential consequences. We hope that our report of the IES phenomenon will motivate further case studies of similar miscitation practices, so that the present article was a puzzle piece that contributes to the acknowledgement of an encompassing problem in academia. Going into more depth to understand a specific case, such case studies complement broader, but necessarily more superficial, surveys such as Cobb et al. (2024).

Apart from undisclosed secondary citations of Townsend and Ashby (1978, 1983), we noted two other types of miscitations. First, even if citing authors have read the relevant paragraphs in Townsend and Ashby (1983), they might not have taken enough time or lack the training to understand them. Some engagement with their work is needed to understand that Townsend and Ashby (1978, 1983) in no way recommended applying the formula to data generally. Our discussion of a comment from an anonymous reviewer in the section “The origin of IES” exemplifies this mistake. Second, Bruyer and Brysbaert (2011) and even Liesefeld and Janczyk (2019) are sometimes cited as support for the validity and/or usefulness of IES, despite their clearly pessimistic evaluation of the measure (e.g., Breitling-Ziegler et al., 2020; Friedrich et al., 2021; Jiao et al., 2024). The rationale might be that these sources have evaluated IES and pointing the readers to them would allow them to form their own critical evaluation of the reported IES results. It appears unlikely that many readers will do that and, in the most benign interpretation, such citations would miss an important nuance of the results (that IES has been invalidated rather than supported), according to Cobb et al.’s (2024) classification.

This is not to say that every researcher should read and understand the original sources for every method they employ in their work. A more extreme (and therefore forgivable) example would be to employ a *t* test without reading the original paper by Student (1908). As there are no criteria yet on where to draw the line, one might argue that methods can be considered sufficiently established to uncritically use them when they are part of a typical psychology curriculum. However, it is precisely the textbook knowledge that seems to result in the most serious misconceptions due to a lack of verifying the sources (Gigerenzer, 2004; Gould, 1988, as cited in Wynn, 2010). In any case, whenever a new method starts to emerge, it should be the responsibility of those who first adopt this method to also critically evaluate and point out the evidence (or the absence of evidence) for its validity. That way, maldevelopments might be caught early on. Such behavior might avoid “establishing” some methods that have never been validated theoretically or empirically to begin with (see also Gigerenzer, 2004), but it would not assure that only perfectly valid methods become mainstream. Rather, we believe that scientific methods must be subject to constant change and challenge. Rather than being uncritically used on the premise that prior users certainly knew what they were doing, even established methods need to be subjected to validation studies (exemplified for combined performance measures, including IES, in Liesefeld & Janczyk, 2019). Existing validations are not necessarily perfect and must therefore undergo regular re-examination (exemplified for combined performance measures in Liesefeld & Janczyk, 2023).

Cobb et al. (2024) discuss in detail what individual authors, reviewers, and editors can do to improve citation practices. The research community could incentivize this commendable behavior by increasing the costs for miscitations. For example, authors can be held responsible by publishing errata for any detected miscitation. This might deter some from taking the shortcut of relying on secondary citations (Klitzing et al., 2018). To the degree that this is not feasible, a compromise would be to encourage the use of secondary (indirect) citations (e.g., “Townsend & Ashby, 1978, as cited in Bruyer & Brysbaert, 2011”). Currently, these seem to be shunned in the academic culture. Even though the American Psychological Association gives clear advice on how to cite indirectly, they also recommend: “Cite secondary sources sparingly – for instance, when the original work is out of print, unavailable, or available only in a language that you do not understand. If possible, as a matter of good scholarly practice, find the primary source, read it, and cite it directly rather than citing a secondary source.” (American Psychological Association, 2020, pp. 273–274). Maybe in part due to this well-intentioned advice, secondary citations may be something that scholars avoid because they are concerned about the judgment of colleagues. Being more open about them could be a step forward, not just because citing the original sources without reading them is even worse but also because disguising where the information came from risks that the next authors do the same thing, thus producing concealed tertiary, quaternary, and quinary citations. The practice of concealed secondary citations may have exacerbated the problems that we have illustrated with the example of IES here.

We therefore call for more honest citation practices. Either incentivize authors to read and understand the primary sources or the community needs to become more accepting of secondary citations. The latter would be based on the understanding that we do not know the authors’ situation and what was possible for them (i.e., whether they have the opportunity to read or the required training to understand the primary sources). At least, secondary citations reflect honesty in the author and maintain the history of information transmission, so that any claims can be verified without laborious detective work by other researchers. An incentive that could be easily implemented by journals is to state explicitly in the author instructions that secondary citations used sparingly are acceptable and will not necessarily be counted against the author, although a careful check of the primary reference would of course always be preferable (American Psychological Association, 2020; Cobb et al., 2024). In terms of the telephone-games analogy, instead of claiming that “Jack’s word was [credentials]” one should more honestly say that “Marc told me that Kate told him that Jack’s word was [credentials].” Even better would be to ask Jack directly – if time allows and Jack is still available.

## Footnotes

^1^
Note that such a pattern does not necessarily mean that there is only an SAT effect and, conversely, the absence of this pattern does not guarantee that there is no SAT effect. More direct empirical markers of such effects are yet to be developed (if possible). In the meantime, such a discrepancy between aggregated RTs and PCs will rightly evoke this suspicion in many readers.

^2^
A simplified version of this formula is usually given as IES = RT/PC. However, this formula leaves many details unspecified, namely (a) that typically only correct RTs are used, (b) that these are averaged across trials, and (c) that IES is calculated for each participant and condition using only data from this participant and condition.

^3^
We analyzed papers (mainly journal articles, preprints, and theses) that were published in 2023 and cited either Townsend and Ashby (1978), Townsend and Ashby (1983), or Bruyer and Brysbaert (2013) according to a Google Scholar search in July 2024. Among the 75 papers using IES, 12 papers mention no discernible reason, six even purport that IES would measure SAT, and only three refer to “the average energy consumed by the system” mentioned by Townsend and Ashby (1983), one of which (Xiao et al., 2023) is a verbatim reproduction of the IES description given by Liesefeld and Janczyk (2019).

^4^
For other similar-looking combined performance measures that either use a different standardization and therefore work less well (Liesefeld & Janczyk, 2023) or are linear transforms of BIS, see Vandierendonck (2017) and Shore et al. (2001).

^5^
Liesefeld and Janczyk (2019) have shown that IES typically largely reflects across-cell variation in mean RTs and, consequently, not much information is gained if it confirms mean RT results.
